# Cytoplasmic translocation of tripartite motif-containing 28 is critical for PRRSV-induced autophagy through promoting Vps34-Beclin1 complex formation

**DOI:** 10.1128/jvi.01133-25

**Published:** 2025-09-24

**Authors:** Meng Chen, Yuna Zhao, Hui An, Qingbing Han, Chenchen Cui, Jun Peng, Yihong Xiao, Gang Wang, Yingli Shang

**Affiliations:** 1 Shandong Provincial Key Laboratory of Zoonoses, College of Veterinary Medicine, Shandong Agricultural University 34734, Taian, Shandong, China; 2 State Key Laboratory for Animal Disease Control and Prevention, Harbin Veterinary Research Institute, Chinese Academy of Agricultural Sciences 111613 , Harbin, China; University of Kentucky College of Medicine, Lexington, Kentucky, USA

**Keywords:** PRRSV, TRIM28, autophagy, CRM1-dependent pathway

## Abstract

Autophagy, as a highly conserved cellular metabolic regulation mechanism, is a double-edged sword and plays multiple roles in viral infections processes. As a member of the Arteriviridae family within the order Nidovirales, the porcine reproductive and respiratory syndrome virus (PRRSV) induces cell autophagy both *in vitro* and *in vivo*. However, the direct or indirect causation of autophagy by PRRSV remains unclear. Identified as an autophagy-related factor, tripartite motif-containing 28 (TRIM28) shows an undefined relationship with autophagy during PRRSV infection. This study investigates the dynamic changes in autophagy and TRIM28 during PRRSV infection, revealing that PRRSV Nsp4 is identified as a key component responsible for the nuclear export of TRIM28 via a CRM1-dependent pathway, promoting the formation of the Vps34-Beclin1 complex and ultimately initiating autophagy. As a host protein, TRIM28 has exerted a certain antiviral effect, but the mechanism is not yet clear. This study provides detailed insight into the mechanism of PRRSV-mediated autophagy for the first time, offering valuable information for understanding the pathogenesis of porcine reproductive and respiratory syndrome.

**IMPORTANCE:**

PRRS is one of the major diseases affecting the global swine industry. Infection with PRRSV can cause respiratory disease in pigs of all ages and reproductive disorders in sows. Therefore, understanding the interaction between PRRSV and host factors may help to develop new antiviral strategies against PRRSV. We found that PRRSV Nsp4 was important for nuclear export of TRIM28 in a CRM1-dependent manner during PRRSV infection. TRIM28 in the cytoplasm increases the formation of VPS34-Beclin1 complex by interacting with Vps34, further initiating autophagy. Hence, our study reveals a novel mechanism of PRRSV-mediated autophagy and provides valuable information for further understanding the pathogenesis of PRRS, which might contribute to the development of novel antiviral drugs.

## INTRODUCTION

Porcine reproductive and respiratory syndrome virus (PRRSV) and equine arteritis virus (EAV) are significant veterinary pathogens within the Arteriviridae family, order Nidovirales (1, 2). PRRSV is the primary agent of porcine reproductive and respiratory syndrome (PRRS), a major economic burden on the global swine industry (3). PRRSV, characterized by a positive-stranded genomic RNA within an enveloped icosahedral viral capsid, includes two distinct species, PRRSV-1 and PRRSV-2, identified since 2017 (4). The PRRSV-2 genome, approximately 15 kb in length, comprises at least 10 open reading frames (ORFs), including ORF1a, ORF1b, ORF2a, ORF2b, ORFs3–7, and ORF5a (5, 6). ORF1a and ORF1b encode 16 viral nonstructural proteins (Nsps), such as Nsp1α, Nsp1β, Nsp2, Nsp2TF, Nsp2N, Nsp3–6, Nsp7α, Nsp7β, and Nsp8–12 (7–12). The roles of these Nsps in the PRRSV life cycle, including their involvement in PRRSV-induced autophagy, have been examined (13–15). However, the specific role of host proteins in PRRSV-induced autophagy remains unclear.

Autophagy, an evolutionarily conserved and highly regulated catabolic process, involves delivering cytoplasmic components to lysosomes for clearance and recycling (16). The formation of an isolation membrane is initiated by Class III phosphatidylinositol 3-kinase, also known as vacuolar protein sorting 34 (Vps34), which converts phosphatidylinositol into phosphatidylinositol-3-phosphate and forms a complex with Vps34/Beclin1 (17). The elongation of the autophagic membrane requires processing by two ubiquitin-like protein-conjugation systems: Atg5–Atg12 and LC3 (18). Autophagy plays diverse physiological roles, including stress adaptation, development, lipid metabolism, degenerative diseases, protection against inflammation, and defense against intracellular pathogens (17). Host-cell/pathogen co-evolution has led to the selection of microorganisms capable of evading or exploiting autophagy. As a positive-sense RNA virus, studies have reported that PRRSV-2 induces autophagy *in vitro* and *in vivo* (13–15). However, the mechanisms by which PRRSV infection induces autophagy are not well understood, necessitating further investigation to elucidate the molecular mechanisms involved.

Tripartite motif-containing 28 (TRIM28), also known as KAP1 or TIF1, was initially identified as a nuclear co-repressor for KRAB domain-containing zinc finger proteins (19). It interacts with these proteins to target specific genomic regions and modulates transcription through interactions with HP1 isoforms (20). Additionally, TRIM28 regulates the initiation and elongation of RNA polymerase II (Pol II)-dependent transcription and participates in cellular activities such as cell differentiation, DNA damage response, tumorigenesis, cytokine production, viral replication, stem cell pluripotency, embryonic development, and autophagy (21). Further evidence suggests TRIM28 also functions independently of gene regulation by serving as a signaling scaffold protein, mediating signal transduction through multiple post-translational modifications (PTMs), including serine/tyrosine phosphorylation, SUMOylation, and acetylation, which coordinately regulate its function and protein abundance (22). As an E3 ubiquitin ligase, TRIM28 promotes PRRSV replication by inhibiting viral protein GP4 ubiquitination (23), enhances SARS-CoV-2 virulence by increasing nucleocapsid protein SUMOylation (24), and promotes NLRP3 inflammasome activation (25). TRIM28 also forms a repressor complex containing heterochromatin protein 1 (HP1), with importin α playing a crucial role in its nuclear delivery and interaction with HP1. The 462–494 amino acid region of TRIM28 serves as a nuclear localization signal overlapping with its HP1-binding site, known as the HP1 box (19). TRIM28 is considered a critical transcriptional co-repressor for autophagy due to its binding to the conserved KRAB repression domain of many transcription factors (26, 27). It has been suggested that TRIM28 promotes porcine epidemic diarrhea virus replication through the inhibition of the JAK-STAT1 pathway mediated by autophagy (28). However, the role of TRIM28 in PRRSV infection-induced autophagy remains unclear and requires further study.

This study investigates the dynamic changes in autophagy and TRIM28 in response to PRRSV infection. The results demonstrate that PRRSV infection induces TRIM28 translocation from the nucleus to the cytoplasm, with PRRSV Nsp4 playing a critical role in the nuclear export of TRIM28 in a CRM1-dependent manner. In the cytoplasm, TRIM28 promotes the formation of the Vps34–Beclin1 complex, initiating the autophagy process. This study reveals a novel mechanism of PRRSV-mediated autophagy and provides valuable insights into the pathogenesis of PRRS.

## RESULTS

### PRRSV infection induced autophagy accompanied by the increase of cytoplasmic TRIM28

**Fig 1:**
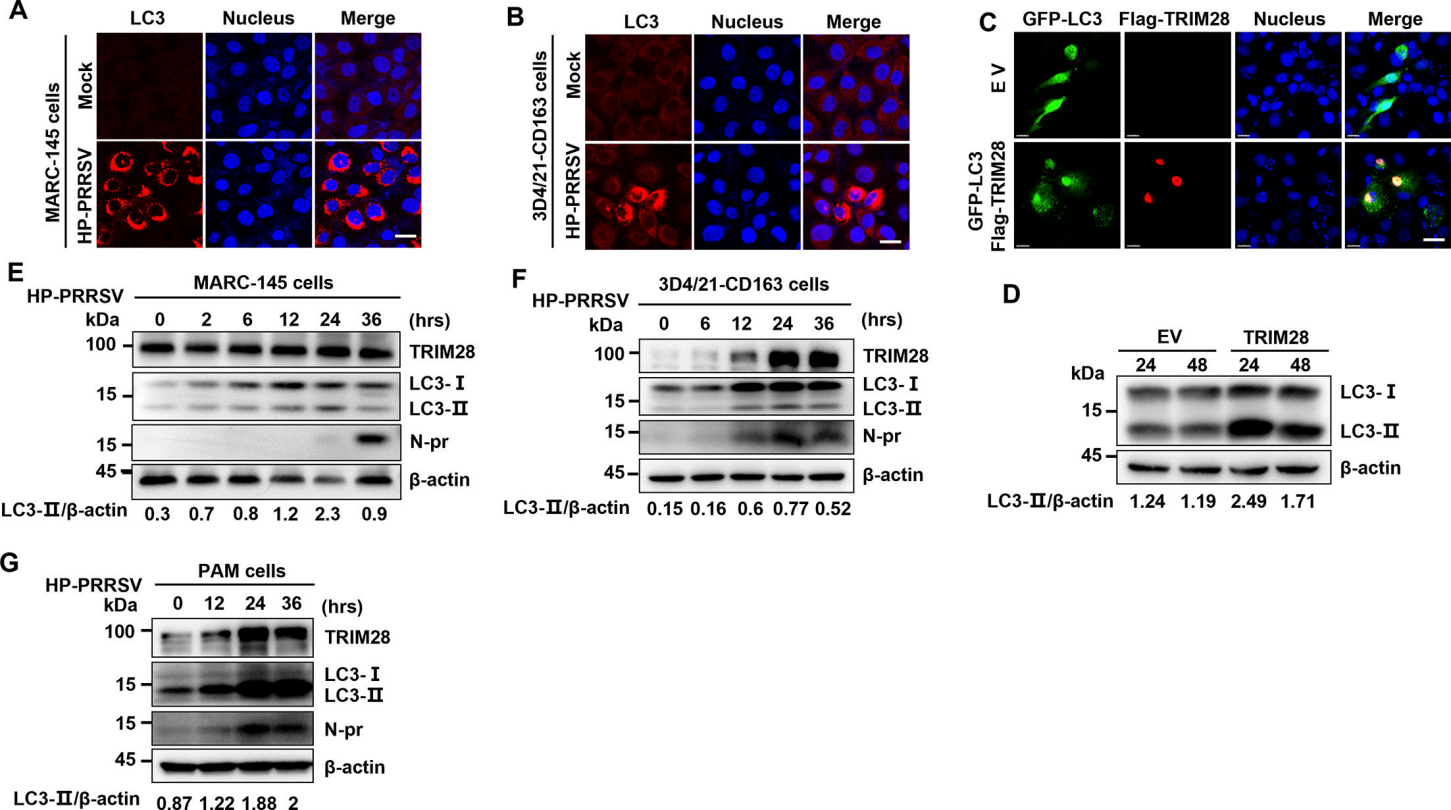
Correlation between PRRSV-induced autophagy and TRIM28. (**A and B**) Confocal microscopy analysis of LC3 puncta in Marc-145 (**A**) and 3D4/21 (**B**) cells infected with HP-PRRSV for 36 h. Scale bars, 20 µm. (**C**) Confocal analysis of LC3 puncta in Marc-145 cells co-transfected with GFP-LC3 and Flag-TRIM28 or an empty vector, 24 h post-transfection. Scale bars, 5 µm. (**D**) Immunoblot analysis of LC3 in NP-40 cell lysates from Marc-145 cells transfected with Flag-TRIM28 or an empty vector for 24 h. (**E through G**) Immunoblot analysis of TRIM28, LC3, and N-Pr in NP-40 cell lysates from Marc-145 (**E**), 3D4/21 (**F**), or PAM (**G**) cells infected with HP-PRRSV (MOI=0.1) for the indicated times.

PRRSV infection induces autophagy both *in vitro* and *in vivo* (29), as evidenced by increased LC3 puncta due to the conversion of LC3I to LC3II, detectable via immunofluorescence staining or Western blot. Verification of PRRSV-induced autophagy employed Marc-145 and 3D4/21-CD163 cell lines. Immunofluorescence observations revealed a significant increase in LC3 puncta in TA-12 and VR2332 strain-infected Marc-145 cells (Fig. 1A; Fig. S1) and 3D4/21-CD163 cells (Fig. 1B; Fig. S1B) at 36 h post-infection (hpi), indicating autophagy induction. The conversion of LC3I to LC3II was confirmed by Western blot from 12 hpi in TA-12-infected Marc-145 cells (Fig. 1E), 3D4/21-CD163 cells (Fig. 1F) and PAM cells (Fig. 1G), as well as VR2332-infected Marc-145 cells (Fig. S1C) and 3D4/21-CD163 cells (Fig. S1D). These results further confirm autophagy in PRRSV-infected cells. TRIM28’s involvement in autophagy (30) prompted an investigation of its dynamic changes. The relationship between TRIM28 and autophagy was confirmed by co-transfecting Marc-145 cells with GFP-LC3 and TRIM28 plasmids. After 24 h, GFP-tagged LC3 puncta increased in co-transfected cells (Fig. 1C), along with LC3I to LC3II conversion (Fig. 1D), demonstrating TRIM28-induced autophagy.

PRRSV-induced TRIM28 changes were detected in Marc-145, 3D4/21-CD163 cells, and porcine alveolar macrophages (PAMs) during TA-12 and VR2332 isolate infections. Samples collected at various time points were treated with cytoplasmic lysate NP-40 and analyzed by Western blot. Both TA-12 and VR2332 infections increased cytoplasmic TRIM28 in Marc-145 cells (Fig. 1E; Fig. S1C), 3D4/21-CD163 cells (Fig. 1F; Fig. S1D), and PAMs (Fig. 1G). Control infections with SEV and EMCV did not increase cytoplasmic TRIM28 (Fig. S1E and F). These cumulative results indicate PRRSV infection induces both autophagy and increased cytoplasmic TRIM28.

### PRRSV infection causes TRIM28 relocalization from the nucleus to the cytoplasm

**Fig 2:**
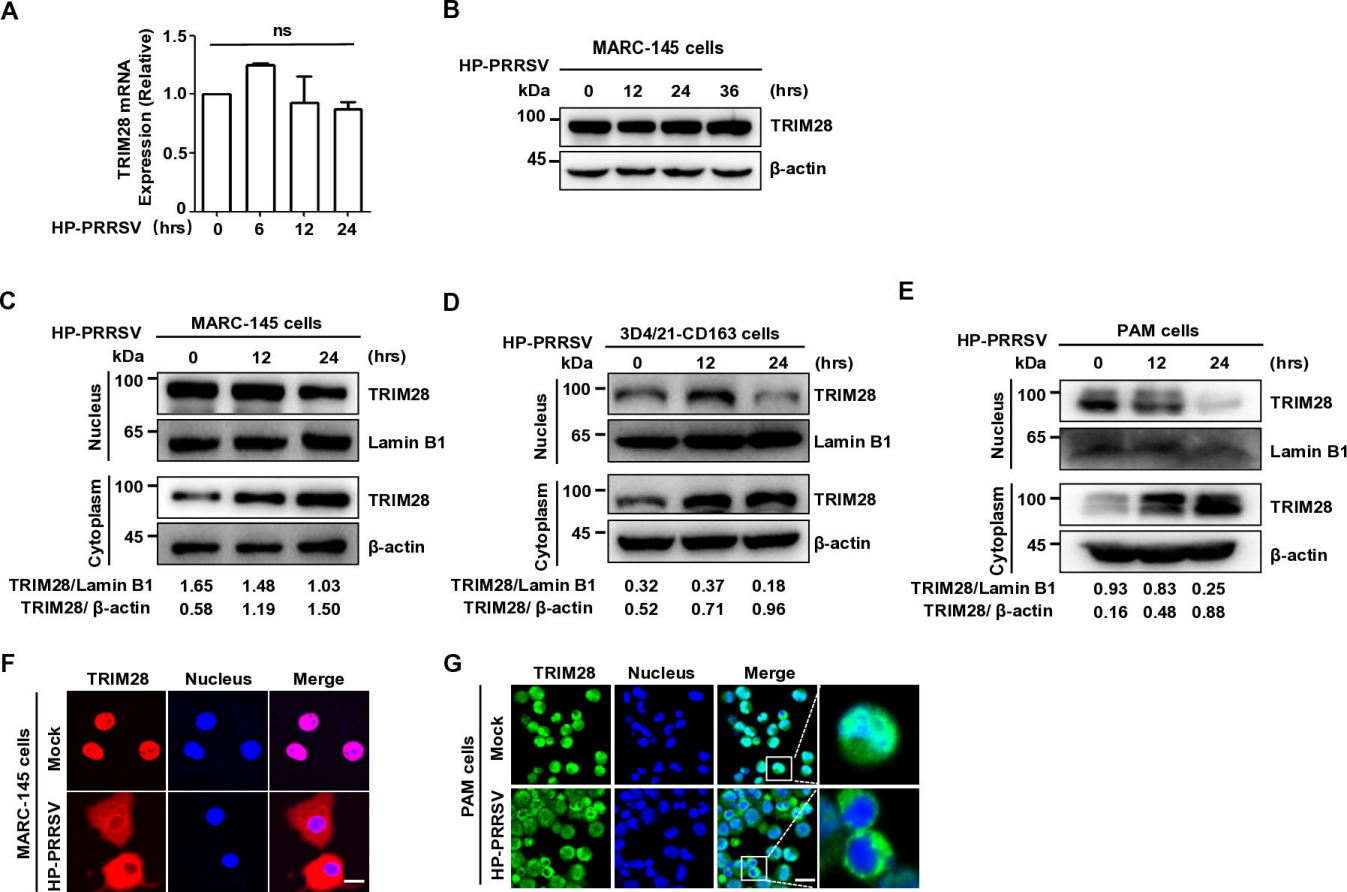
Transfer of TRIM28 from the nucleus to the cytoplasm under PRRSV infection. (**A**) qPCR analysis of TRIM28 in Marc-145 cells infected with HP-PRRSV (MOI=0.1) for the indicated times. (**B**) Immunoblot analysis of TRIM28 in whole-cell lysates of Marc-145 cells infected with HP-PRRSV (MOI=0.1) for the indicated times. (**C through E**) Immunoblot analysis of TRIM28 in cytoplasmic and nuclear fractions of Marc-145 (**C**), 3D4/21 (**D**), and PAM (**E**) cells infected with HP-PRRSV for the indicated time points. (**F and G**) Immunofluorescence detection of TRIM28 localization in Marc-145 cells (**F**) (scale bars, 5 µm) and PAM cells (**G**) infected with HP-PRRSV (MOI=0.1) for 24 h (scale bars, 20 µm).

TRIM28, known as a transcription regulator localized in the nucleus (31), increases in the cytoplasm following PRRSV infection. To clarify the origin of cytoplasmic TRIM28 during PRRSV infection, mRNA levels of TRIM28 were analyzed. Total RNA extracted at various time points was analyzed by qRT-PCR, showing no significant changes in TRIM28 gene levels in Marc-145 and 3D4/21-CD163 cells infected with TA-12 (Fig. 2A) or VR2332 (Fig. S2A) compared to the control group. This suggests that PRRSV infection affects TRIM28 localization rather than its quantity. Using a sodium dodecyl sulfate (SDS) lysis buffer, the total TRIM28 protein content in PRRSV-infected cells was examined. Western blot analysis revealed no changes in total TRIM28 protein content during TA-12 (Fig. 2B) and VR2332 (Fig. S2B) infections, indicating that total TRIM28 levels remain unchanged during PRRSV infection.

The localization of TRIM28 in PRRSV-infected cells was investigated using nucleocytoplasmic isolation assays and immunofluorescence analysis. Western blot analysis demonstrated that both TA-12 and VR2332 (MOI=0.1) infections decreased nuclear TRIM28 protein and increased cytoplasmic TRIM28 protein in Marc-145 cells (Fig. 2; Fig. S2C), 3D4/21-CD163 cells (Fig. 2D), and PAMs (Fig. 2E). Immunofluorescence staining confirmed TRIM28 relocalization from the nucleus to the cytoplasm in PRRSV-infected Marc-145 cells (Fig. 2F) and PAMs (Fig. 2G). These findings demonstrate PRRSV-induced TRIM28 relocalization from the nucleus to the cytoplasm *in vitro*.

### Nsp4 contributes to the TRIM28 redistribution mediated by the CRM1 pathway

**Fig 3:**
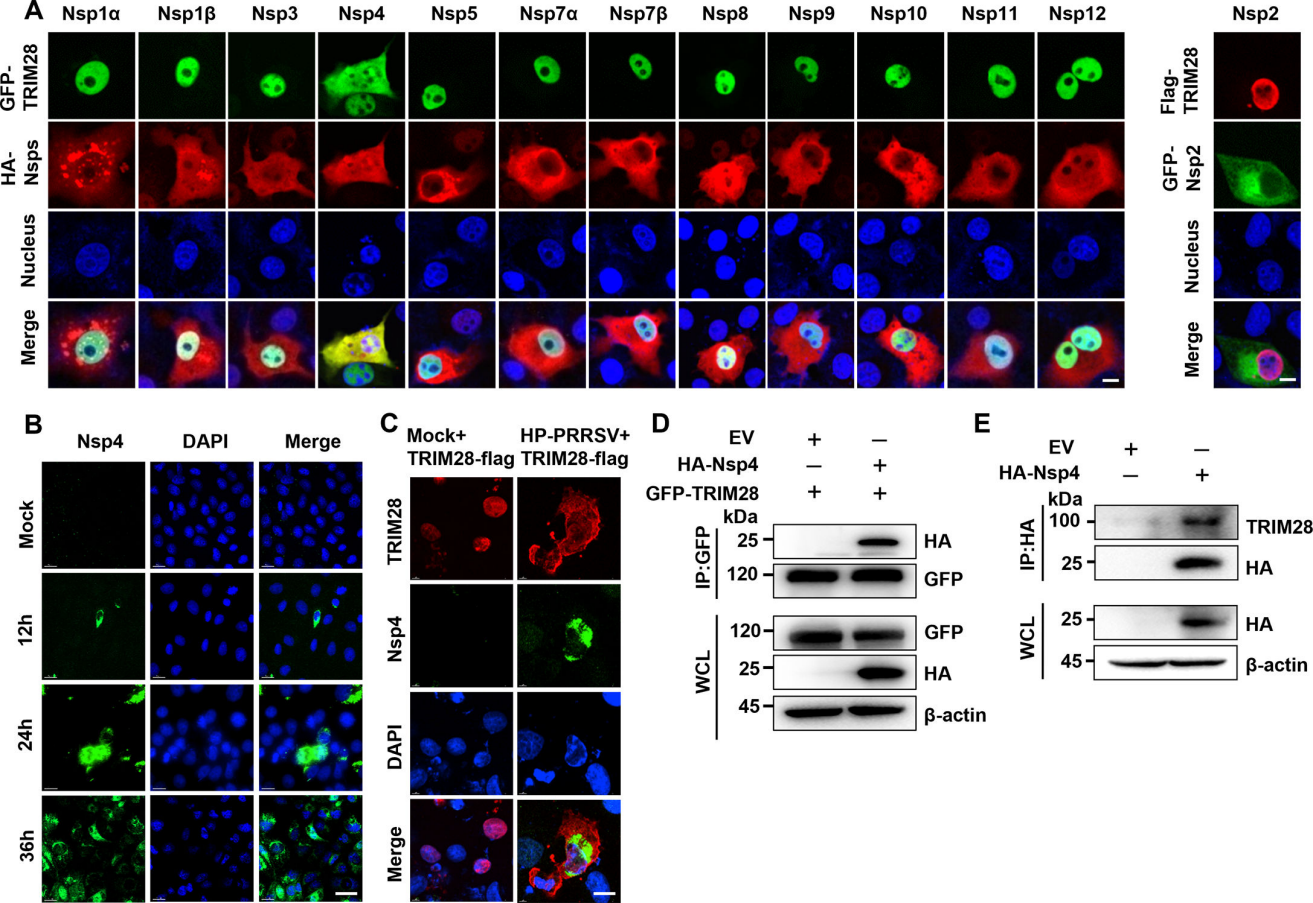
TRIM28 primarily colocalizes with PRRSV Nsp4 in the cytoplasm. (**A**) Colocalization of GFP-TRIM28 (Flag-TRIM28) with HA-tagged PRRSV nonstructural protein (GFP-Nsp2) in Marc-145 cells, examined by immunofluorescence confocal microscopy. Nuclei were stained with DAPI. Scale bars, 5 µm. (**B**) NSP4 was stained and localized in Marc-145 cells infected with HP-PRRSV (MOI=0.1) using anti-NSP4 antibodies within the specified time, and the nuclei were stained with DAPI. Scale bars, 20 µm. (**C**) After transfecting TRIM28 into MARC-145 cells for 24 h, infected with HP-PRRSV (MOI=0.1), colocalization of both was detected 24 h later using anti-Flag antibody and anti-NSP4 antibody. Nuclei were stained with DAPI. Scale bars, 5 µm. (**D and E**) HEK-293T cells co-transfected with TRIM28 and Nsp4 (**D**) or transfected with Nsp4 alone (**E**). After 24 h, cell lysates were precipitated with an anti-HA monoclonal antibody in conjunction with protein A/G PLUS-Agarose beads and analyzed by Western blotting using anti-Flag and anti-HA antibodies.

During PRRSV infection, Nsps facilitate the nuclear translocation of host cell proteins. For instance, Nsp9 interacts with pRb, relocating it from the nucleus to the cytoplasm (32), while Nsp2 and Nsp10 bind with DDX18, similarly transferring it to the cytoplasm (33). To investigate the role of Nsps in TRIM28 relocalization, plasmids encoding PRRSV Nsps were constructed (Fig. S3A and B). Constructs such as pEGFP-C1-TRIM28 and HA-tagged Nsp-expressing pCAGGS (Nsp1α, Nsp1β, Nsp3, Nsp4, Nsp5, Nsp6, Nsp7α, Nsp7β, Nsp8, Nsp9, Nsp10, Nsp11, and Nsp12), or pCDNA3.0-Flag-TRIM28 and pEGFP-C1-Nsp2 were co-transfected into Marc-145 or Hela cells and analyzed via confocal microscopy 24 h post-transfection. Results indicated that only Nsp4 facilitated the cytoplasmic relocalization of TRIM28 in both Marc-145 (Fig. 3A) and HeLa cells (Fig. S3C), with TRIM28 colocalizing with Nsp4. Polyclonal antibodies (pAb) against PRRSV Nsp4 were used to detect the localization of Nsp4 after TA-12 virus infected Marc-145 cells (34). Immunofluorescence staining showed that Nsp4 could be localized in both nucleus and cytoplasm (Fig. 3B), and at the same time, Nsp4 and TRIM28 were colocalized in the cytoplasm in TA-12 infected cells overexpressing TRIM28 (Fig. 3C). This interaction was confirmed in HEK-293T cells through co-transfection, Co-IP using GFP antibodies 24 h post-transfection, and subsequent Western blotting, revealing the presence of both Nsp4 and TRIM28 in the Co-IP product (Fig. 3D). Furthermore, the interaction between Nsp4 and endogenous TRIM28 was corroborated by Nsp4 transfection alone in HEK-293T cells (Fig. 3E). Collectively, these findings suggest that the Nsp4-TRIM28 interaction redistributes TRIM28 from the nucleus to the cytoplasm, decreasing its nuclear abundance.

**Fig 4:**
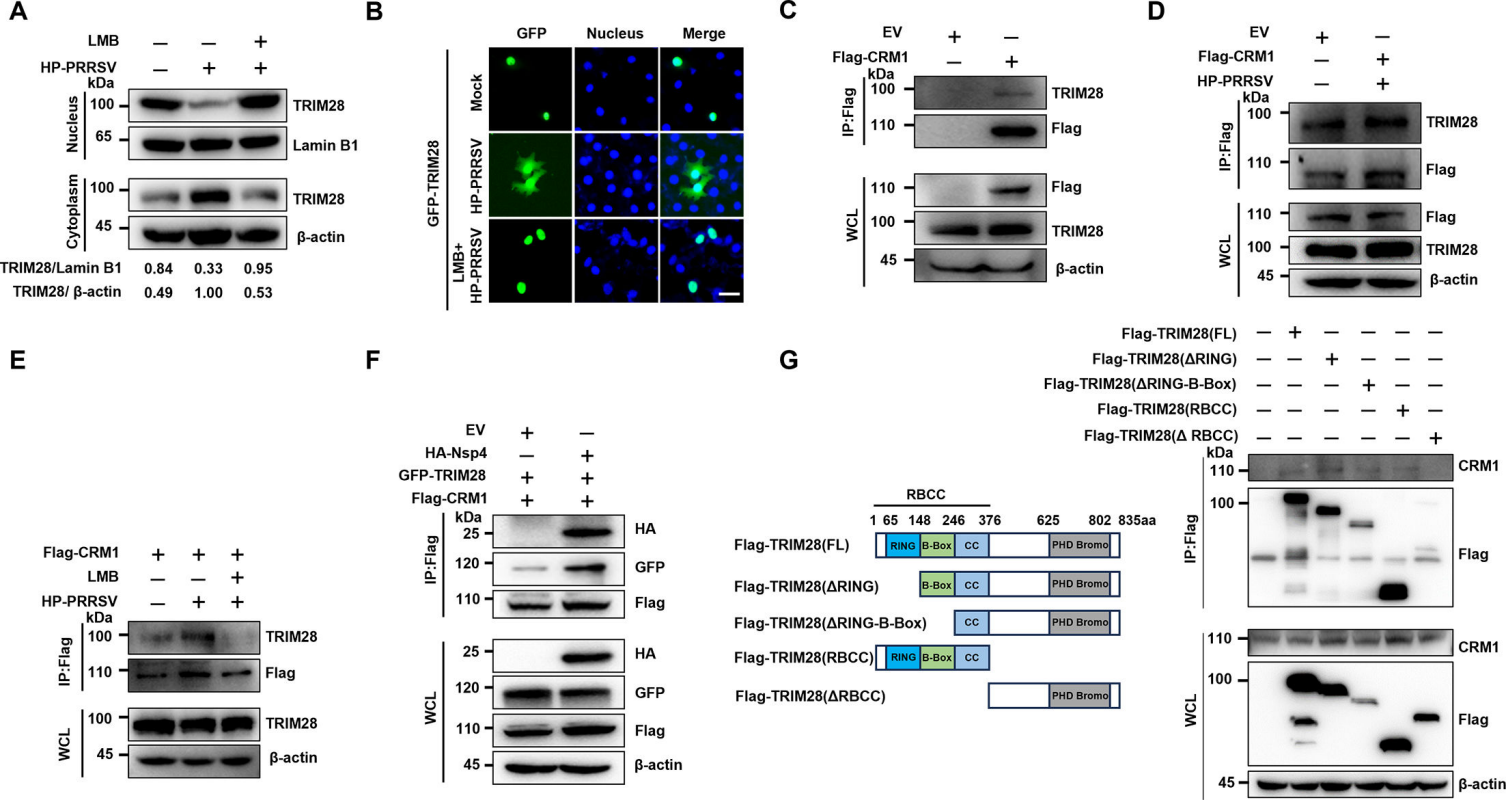
PRRSV-induced nuclear export of TRIM28 depends on CRM1. (**A**) Immunoblot analysis of TRIM28 in cytoplasmic and nuclear fractions of Marc-145 cells infected with HP-PRRSV (MOI=0.1) for 24 h, or treated with 15 nM LMB for 2 h before infection. (**B**) Confocal analysis of Marc-145 cells transfected with GFP-TRIM28 plasmid, infected with HP-PRRSV (MOI=0.1) for 24 h, or treated with 15 nM LMB for 2 h before infection, 24 h post-transfection. scale bars, 10 µm. (**C**) Immunoblot analysis of Flag-CRM1 and TRIM28 in immunoprecipitated and whole-cell lysates of HEK-293T cells. (**D**) Immunoblot analysis of Flag-CRM1 and TRIM28 in immunoprecipitated and whole-cell lysates of Marc-145 cells infected with HP-PRRSV (MOI=0.1) or untreated for 24 h. (**E**) Immunoblot analysis of Flag-CRM1 and TRIM28 in immunoprecipitated and whole-cell lysates of Marc-145 cells infected with HP-PRRSV (MOI=0.1) for 24 h, or treated with 15 nM LMB for 2 h before infection. (**F**) Immunoblot analysis of Flag-CRM1, GFP-TRIM28, and HA-Nsp4 in immunoprecipitated and whole-cell lysates of HEK-293T cells. (**G**) Schematic representation of TRIM28 (WT) and its truncation mutants (left), and co-immunoprecipitation (Co-IP) analysis of CRM1 interaction with Flag-TRIM28 (WT) and TRIM28 truncation mutants in HEK-293T cells (right).

Next, the dependence of TRIM28 transport on the nuclear export receptor CRM1 was investigated. As a key nuclear export receptor, CRM1 mediates protein nucleation, and its inhibitor, Leptomycin B (LMB), binds to CRM1, causing its redistribution to the cytoplasm and inhibiting nuclear import (35, 36). The role of CRM1 during PRRSV infection was examined in Marc-145 cells treated with LMB. Western blotting and immunofluorescence analyses revealed that LMB treatment prevented TRIM28 translocation from the nucleus to the cytoplasm in Marc-145 cells (Fig. 4A and B), indicating that CRM1 is involved in PRRSV-induced TRIM28 transfer. To further confirm the interaction between CRM1 and TRIM28, Flag-CRM1 plasmids were transfected into HEK-293T cells, and Co-IP using Flag antibodies demonstrated the presence of both CRM1 and TRIM28 in the Co-IP product (Fig. 4C), suggesting an interaction. Additionally, dynamic changes in CRM1 and TRIM28 interaction post-TA-12 infection were analyzed. Flag-CRM1 transfection followed by PRRSV inoculation in Marc-145 cells showed upregulation of Flag-CRM1 and TRIM28 (Fig. 4D). However, LMB treatment resulted in the absence of TRIM28 despite CRM1 still being present in the Co-IP product post-PRRSV infection (Fig. 4E). These findings confirm that PRRSV-induced TRIM28 redistribution is CRM1-dependent.

Given Nsp4’s role in TRIM28 redistribution, its involvement in PRRSV-induced TRIM28 relocation via the CRM1 pathway was further examined. HA-Nsp4, GFP-TRIM28, and Flag-CRM1 plasmids were co-transfected into HEK-293T cells to determine Nsp4’s contribution to the TRIM28-CRM1 interaction. Co-immunoprecipitation (Co-IP) with HA antibodies and subsequent Western blotting revealed the co-presence of Nsp4, TRIM28, and CRM1 in the Co-IP product, indicating that Nsp4 enhances the interaction between TRIM28 and CRM1 (Fig. 4F). To identify the functional domain responsible for this interaction, plasmids encoding full-length or truncated TRIM28 were generated. Co-IP with Flag antibodies and Western blotting showed that Flag-TRIM28 co-precipitated with CRM1, with the RBCC domain identified as key to this interaction (Fig. 4G). Collectively, these data demonstrate that PRRSV Nsp4-induced transfer of TRIM28 from the nucleus to the cytoplasm is CRM1-dependent, with the RBCC domain playing a crucial role.

### PRRSV infection-induced autophagy depends on TRIM28

**Fig 5:**
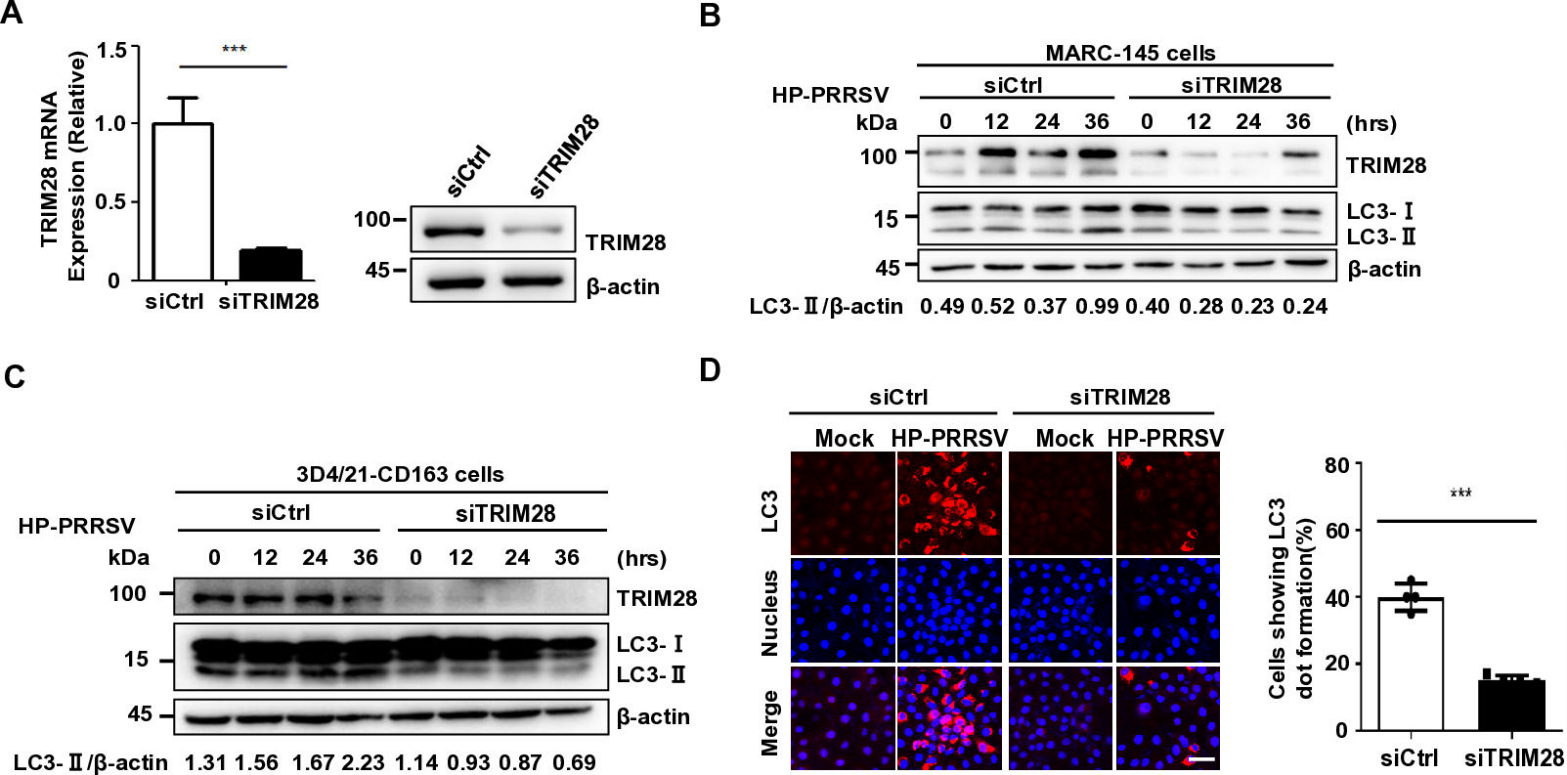
TRIM28 knockdown decreases PRRSV infection-mediated autophagy. (**A**) qPCR analysis of TRIM28 knockdown (left) and immunoblot analysis of Marc-145 cells transfected with negative control (NC) or TRIM28 siRNA (right). (**B**) Immunoblot analysis of TRIM28 and LC3 in NP-40 cell lysates of control or TRIM28 knockdown Marc-145 cells infected with HP-PRRSV (MOI=0.1) for the indicated times. (**C**) Immunoblot analysis of TRIM28 and LC3 in whole-cell lysates of control or TRIM28 knockdown 3D421–CD163 cells infected with HP-PRRSV (MOI=0.1) for the indicated times. (**D**) Confocal analysis of LC3 puncta in control or TRIM28 knockdown Marc-145 cells infected with HP-PRRSV (MOI=0.1) for 36 h. LC3 dot formation in control or TRIM28 knockdown Marc-145 cells. Scale bars, 20 µm. ***P <0.001 (Student’s *t*-test).

PRRSV infection-induced autophagy, coupled with TRIM28 transport, suggests a role for TRIM28 in PRRSV-induced autophagy. To determine whether PRRSV-induced autophagy relies on TRIM28, TRIM28 was knocked down in Marc-145 and 3D4/21 cells using siRNA targeting TRIM28, which effectively inhibited its transcription and expression (Fig. 5A). These cells, along with control siRNA cells, were inoculated with TA-12 at 0.1 MOI. In TRIM28 knockdown cells, PRRSV infection did not induce the conversion of LC3I to LC3II at 12, 24, and 36 h post-infection in both Marc-145 and 3D4/21 cells, as shown by Western blot analysis (Fig. 5B and C). Immunofluorescence analysis also revealed a significant reduction in LC3 spots in Marc-145 cells following TRIM28 knockdown (Fig. 5D). These findings suggest that inhibiting TRIM28 expression impedes PRRSV-induced autophagy. Collectively, this evidence demonstrates that PRRSV-induced autophagy is dependent on TRIM28 participation.

### TRIM28 mediates PRRSV-induced autophagy by interacting with VPS34.

**Fig 6:**
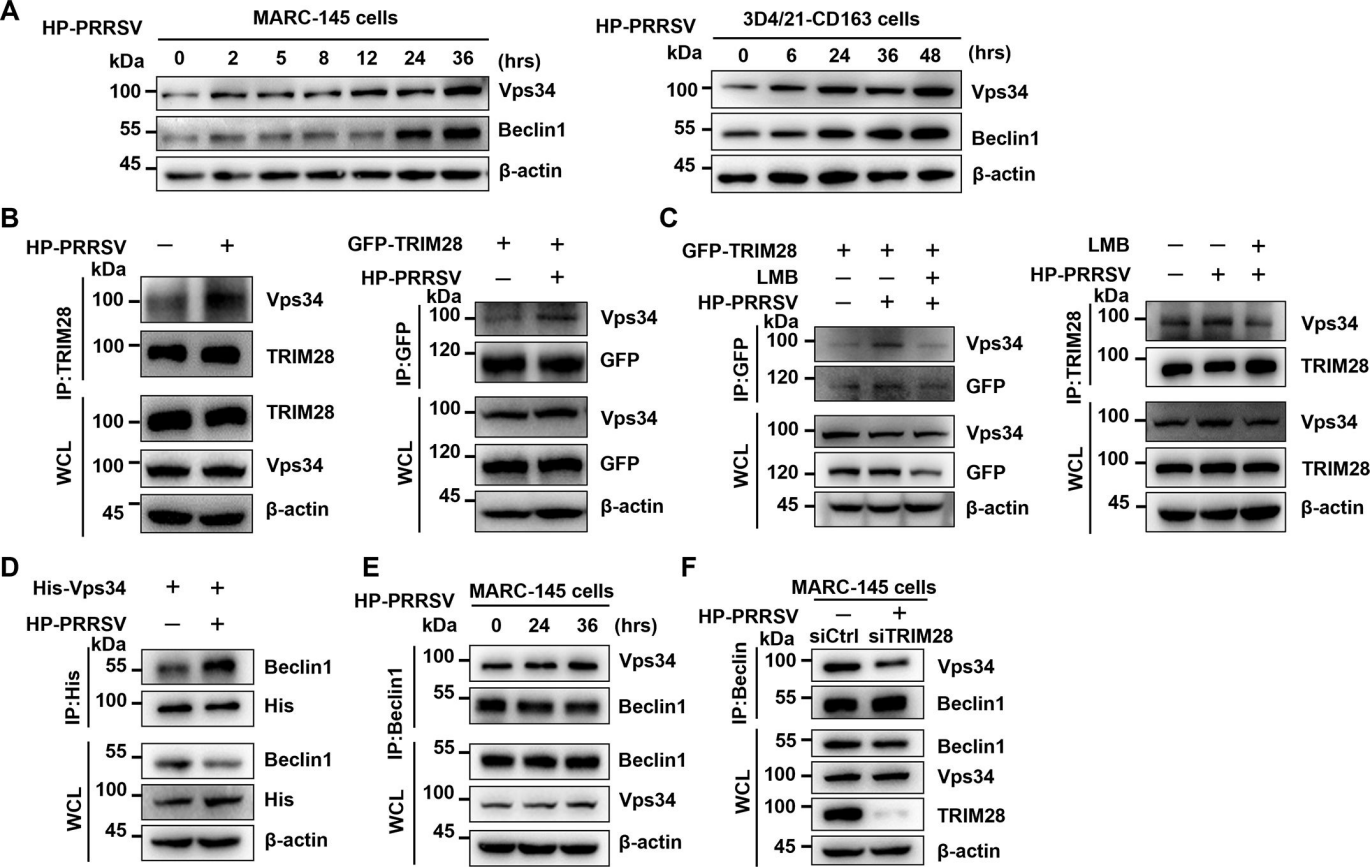
TRIM28 promotes PRRSV-induced autophagy by interacting with VPS34 and Beclin1. (**A**) Immunoblot analysis of VPS34 and Beclin1 in NP-40 cell lysates of Marc-145 (left) and 3D421-CD163 (right) cells infected with HP-PRRSV (MOI=0.1) for the indicated times. (**B**) Immunoblot analysis of VPS34 and TRIM28 (upper)/GFP-TRIM28 (lower) in immunoprecipitated and whole-cell lysates of Marc-145 cells infected with HP-PRRSV (MOI=0.1) or untreated for 24 h. (**C**) Immunoblot analysis of VPS34 and GFP-TRIM28 (left) / TRIM28 (right) in immunoprecipitated and whole-cell lysates of Marc-145 cells infected with HP-PRRSV (MOI=0.1), treated with 15 nM LMB before infection, or untreated for 24 h. (**D**) Immunoblot analysis of Beclin1 in His-VPS34 immunoprecipitates from Marc-145 cells infected with HP-PRRSV (MOI=0.1) or untreated for 36 h. (**E**) Immunoblot analysis of Beclin1 and VPS34 in immunoprecipitates from Marc-145 cells infected with HP-PRRSV (MOI=0.1) for the indicated times. (**F**) Immunoblot analysis of Beclin1 and VPS34 in immunoprecipitates from control or TRIM28 knockdown Marc-145 cells infected with HP-PRRSV (MOI = 0.1) for 36 h.

Given that PRRSV infection-induced autophagy depends on TRIM28, the role of TRIM28 as an autophagy regulator interacting with autophagy-associated proteins was further investigated. Vps34, also known as PI3K catalytic subunit type III (PI3KC3), is a central autophagy protein that forms a complex with Beclin1, Vps15, and ATG14L (37–39). During TA-12 infection in Marc-145 and 3D4/21 cells, Western blot analysis showed upregulation of Vps34 and Beclin1 (Fig. 6A). To determine if PRRSV infection affects the interaction between TRIM28 and Vps34, Co-IP using TRIM28 pAb antibodies at 24 h post-infection revealed PRRSV-induced upregulation of Vps34. Similarly, GFP-TRIM28 transfected Marc-145 cells inoculated with TA-12 at 0.1 MOI showed increased Vps34 levels via Co-IP with GFP antibodies and subsequent Western blotting (Fig. 6B). These findings suggest that PRRSV infection enhances the interaction between TRIM28 and Vps34. Additionally, the effect of CRM1-mediated TRIM28 nucleation on the TRIM28-Vps34 interaction was examined. GFP-TRIM28 transfected Marc-145 cells were inoculated with TA-12 at 0.1 MOI or treated with LMB before TA-12 inoculation. Co-IP with anti-GFP antibodies showed that PRRSV infection enhanced the interaction between GFP-TRIM28/endogenous TRIM28 and Vps34, while LMB treatment weakened this interaction (Fig. 6C). Given the importance of the Vps34-Beclin1 complex in autophagy induction (40), the interaction between Vps34 and Beclin1 during PRRSV infection was also investigated.

The interaction between Vps34 and Beclin1 was confirmed through transfection of Vps34 in Marc-145 cells, followed by Co-IP using antibodies against Beclin1 to identify the interaction between endogenous Vps34 and Beclin1. The results indicated that the interaction between Vps34 and Beclin1 was significantly enhanced after PRRSV infection (Fig. 6D and E). Additionally, Co-IP assays demonstrated that Vps34 and Beclin1 interact weakly following TRIM28 knockdown in Marc-145 or 3D4/21 cells (Fig. 6F). These data reveal that TRIM28 directly interacts with Vps34 and modulates the Vps34-Beclin1 interaction to induce autophagy.

### TRIM28 regulates PRRSV replication

**Fig 7:**
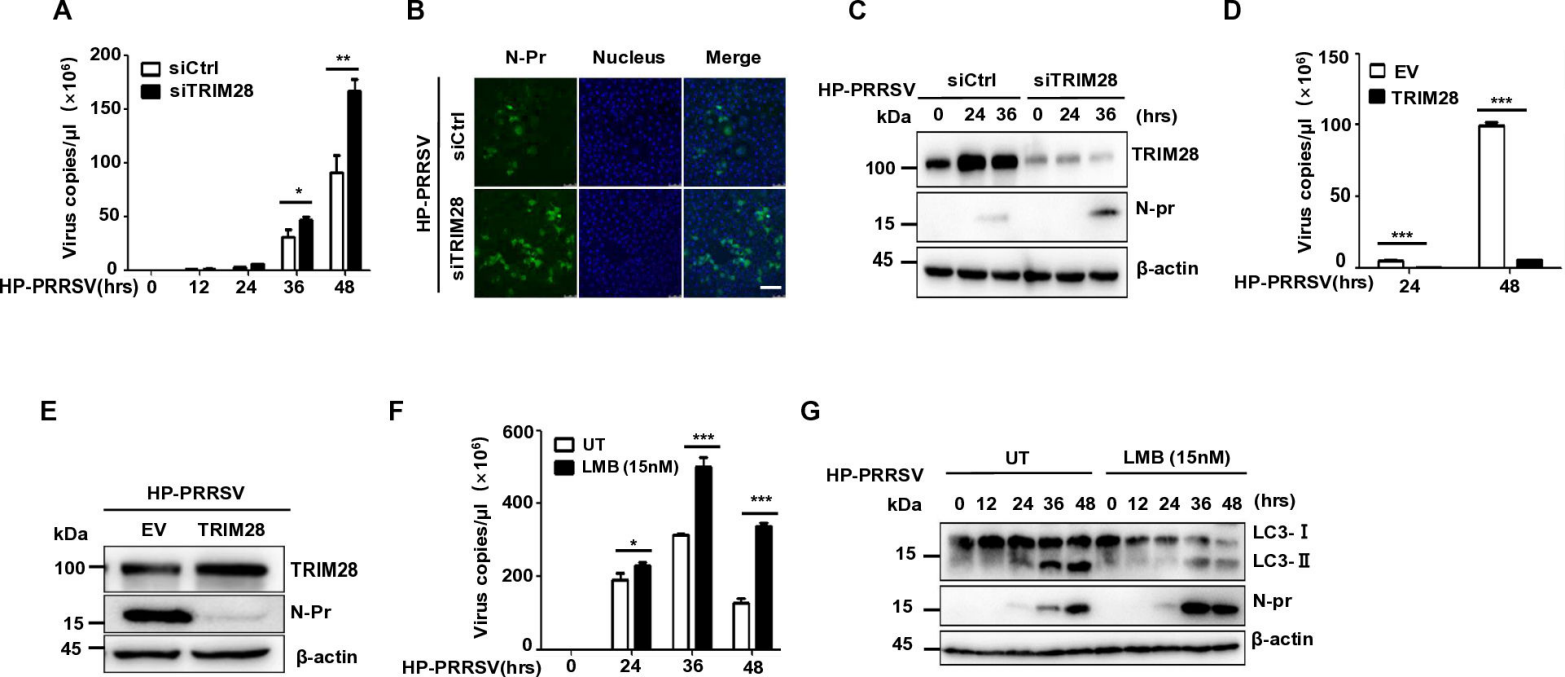
TRIM28 regulates PRRSV replication. (**A**) qPCR analysis of virus copies in control or TRIM28 knockdown Marc-145 cells infected with HP-PRRSV (MOI=0.05) for indicated times. (**B**) Immunofluorescence analysis of N protein in control or TRIM28 knockdown Marc-145 cells infected with HP-PRRSV (MOI=0.1) for 36 h. Scale bars, 100 µm. (**C**) Immunoblot analysis of TRIM28 and N protein in control or TRIM28 knockdown Marc-145 cells infected with HP-PRRSV (MOI=0.1) for 36 h. (**D**) qPCR analysis of virus copies in Marc-145 cells which were transfected with TRIM28 or control empty vector. 24 h post transfection, infected with HP-PRRSV (MOI=0.1) for 24 and 48 h. (**E**) Marc-145 cells were transfected with TRIM28 or empty vector. 24 h post transfection, infected with HP-PRRSV (0.1 MOI) for 48 h, immunoblot analysis of TRIM28 and N protein. Data are pooled from three independent experiments (**A and D**). (**F**) qPCR analysis of the viral copy number of the control or Marc-145 cells (MOI=0.1) with LMB (15 nM) added at the indicated time. (**G**) At the designated time after Marc-145 cell infection with the virus (MOI=0.1), LMB (15 nM) treatment was added, and immunoblot analysis was performed on LC3I/II and N protein.

Autophagy is closely related to viral replication, and whether TRIM28-mediated autophagy induction affects viral replication was also examined. TRIM28, a known transcriptional corepressor for KRAB domain-containing zinc finger transcription factors (41), was targeted via knockdown at Marc-145 cells. The results showed knockdown of TRIM28 significantly increased PRRSV copy numbers after PRRSV infection, particularly at 48 h (Fig. 7A). Consistently, immunofluorescence revealed elevated levels of the viral N protein at 36 hpi following TRIM28 knockdown (Fig. 7B), and confirmed by western blotting (Fig. 7C). Conversely, TRIM28 overexpression reduced viral copy numbers at 24 and 48 hpi (most notably at 48 h; Fig. 7D) and decreased N protein levels (Fig. 7E). To determine whether TRIM28’s nuclear translocation regulates PRRSV replication, we treated Marc145 cells with LMB, a CRM1 inhibitor that blocks nuclear export. LMB treatment increased viral copy numbers (especially at 36 hpi; Fig. 7F) and enhanced N protein expression (Fig. 7G), indicating that nuclear retention of TRIM28 suppresses PRRSV. Collectively, these data demonstrate that TRIM28 expression and nuclear localization inhibit PRRSV replication.

## DISCUSSION

PRRSV infection-induced autophagy has been studied both *in vitro* and *in vivo* (29, 42, 43), yet the mechanisms of virus-host interactions remain incompletely understood. This study found that during PRRSV infection, Nsp4 induces TRIM28 redistribution from the nucleus to the cytoplasm via the CRM1 pathway, forming a complex with VPS34 and Beclin1 to trigger autophagy. The output of this TRIM28 is likely a host defense mechanism. This study first elucidates the potential mechanism of autophagy induction by PRRSV infection and the dynamic changes of TRIM28, providing an understanding of the host’s antiviral mechanisms.

TRIM28 has been proven to be an autophagy-related factor (30), relocating from the nucleus to the cytoplasm during PRRSV infection. PRRSV Nsps are known to mediate the nuclear translocation of host cell proteins during infection (32, 33). Building on these studies, it was hypothesized that Nsps contribute to TRIM28 translocation during PRRSV infection. Plasmids for PRRSV Nsps and TRIM28 were constructed, and Nsp4 was identified as the only Nsp interacting with TRIM28 in the cytoplasm. Nsp4, the primary proteinase in EAV, possesses chymotrypsin-like serine protease activity (44, 45). During PRRSV replication, Nsp4 localizes in both the cytoplasm and nucleus (6, 46), which supports its colocalization with TRIM28 in the cytoplasm. These results suggest that Nsp4 may facilitate nuclear translocation, warranting further investigation.

The export of large nuclear proteins (>50 kDa) relies on the export adapter protein Nmd3, which provides a nuclear export signal (NES). The leucine-rich NES is recognized by the export receptor CRM1, facilitating passage through nuclear pore complexes in the nuclear membrane (36, 47, 48). It has not been previously reported whether TRIM28 export requires transport receptors. In this study, Western blotting and immunofluorescence results showed that TRIM28 translocation from the nucleus to the cytoplasm occurs alongside up-regulated CRM1 during PRRSV infection, suggesting a CRM1-dependent nuclear export pathway. To further investigate CRM1’s role in TRIM28 translocation after PRRSV infection, the CRM1 inhibitor LMB was used. LMB, a 540 Da polyketide, binds with CRM1, causing its redistribution from the nucleus to the cytoplasm (35, 36). Marc-145 cells treated with LMB 3 h before PRRSV infection exhibited CRM1 redistribution from the nucleus to the cytoplasm. The results indicated that LMB treatment retained TRIM28 almost entirely in the nucleus, confirming TRIM28 translocation via the CRM1-dependent nuclear export pathway during PRRSV infection.

Another interesting observation is the simultaneous occurrence of TRIM28 translocation and autophagy in PRRSV-infected Marc-145 cells. TRIM28’s participation in autophagy has been reported in glioblastoma (49), where ubiquitination and degradation of AMP-activated protein kinase (AMPK) by the cancer-specific MAGE-A3/6-TRIM28 ubiquitin ligase inhibit autophagy in cancer cells (30). These observations raise the question of whether autophagy is induced directly by PRRSV infection or as a result of TRIM28 export. To investigate the relationship among PRRSV infection, TRIM28 translocation, and autophagy, siRNA targeting TRIM28 was used in Marc-145 and 3D4/21 cells. The conversion of LC3I to LC3II did not occur in these cells, demonstrating that PRRSV-induced TRIM28 translocation contributes to autophagy. During autophagy, the formation of double-membrane autophagosomes requires a complex formed by Vps34 bound to Beclin1, Vps15, and ATG14L (37–39). PRRSV infection enhanced the interaction between Vps34 and Beclin1, which was weakened by knocking down TRIM28 in Marc-145 or 3D4/21 cells and by LMB treatment. This finding provides evidence that PRRSV-induced autophagy depends on cytoplasmic TRIM28 participation.

TRIM family members can restrict viral replication either directly or by activating innate immune responses (50). Conversely, viruses may encode antagonists to subvert TRIM proteins, thereby enhancing viral replication (23, 51, 52). TRIM28 has long been recognized as a transcriptional repressor, and its inhibitory role in gene expression has profound implications for viral transcription and replication (53, 54). Accumulating evidence indicates that TRIM28 effectively suppresses the transcription of various herpesviruses, including KSHV, HCMV, and EBV (55–57). Notably, a recent study revealed that TRIM28 functions as a restriction factor for prototype foamy virus replication through two distinct mechanisms: mediating H3K9 trimethylation and promoting degradation of the viral transactivator Tas (58). In this study, we demonstrated that TRIM28 overexpression suppresses PRRSV replication, whereas TRIM28 knockdown or inhibition of its nuclear export (via LMB treatment) enhances viral replication. Given its well-established role in transcriptional suppression, we propose that TRIM28 likely modulates PRRSV infection by inhibiting viral gene expression.

**Fig 8:**
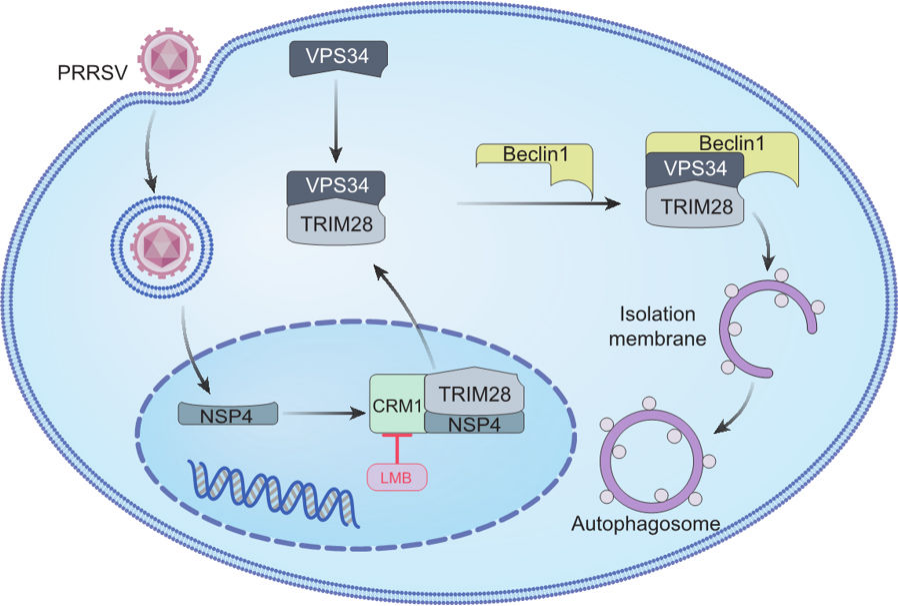
Proposed model for autophagosome formation in Marc-145 cells during PRRSV infection. PRRSV Nsp4 induces TRIM28 transfer from the nucleus to the cytoplasm, where it interacts with VPS34. The TRIM28-VPS34 complex binds to Beclin1, promoting phagophore formation. As the membrane expands, it encloses cytosolic cargos to form the autophagosome.

In conclusion, this study elucidates that PRRSV infection induces autophagy through the translocation of TRIM28 from the nucleus to the cytoplasm, where it forms a complex with VPS34 and Beclin1. In addition, PRRSV Nsp4 was identified as a key factor that facilitates the nuclear export of TRIM28 via a CRM1-dependent pathway (Fig. 8). As a member of the Arteriviridae family within the Nidovirales order, PRRSV is the primary causative agent of PRRS, which poses a significant challenge in veterinary medicine. This investigation is the first to detail the mechanism of PRRSV-mediated autophagy, offering valuable insights into the pathogenesis of PRRS and potentially other Nidovirales members, such as SARS-CoV-2, porcine epidemic diarrhea virus, and infectious bronchitis virus.

## MATERIALS AND METHODS

### Cells and viruses

Porcine alveolar macrophage (PAM) cells were cultured in RPMI-1640 medium (Gibco, USA) supplemented with 10% (V/V) fetal bovine serum (FBS, Gibco). The 3D4/21-CD163 PAM cell line, HEK-293T cells, HeLa cells, and Marc-145 cells were maintained in Dulbecco’s modified Eagle’s medium (DMEM, Gibco) with 10% fetal bovine serum (FBS, BI), 100 U/ml penicillin, and 100 µg/ml streptomycin sulfate in a humidified incubator at 37°C and 5% CO_2_. The highly pathogenic PRRSV TA-12 strain (GenBank accession no. HQ416720) and the classical PRRSV VR2332 strain (GenBank accession no. EF536003.1) served as viral inoculum, with titers of 10^7.67^ TCID^50^/mL and 10^6.37^ TCID^50^/mL, respectively. EMCV and SeV were utilized as controls.

### Plasmid construction and transient transfections

The pcDNA6.2/N-EmGFP-DEST-TRIM28 plasmid was acquired from Addgene. The pCDNA3.0-Flag-TRIM28 plasmid was engineered into the pCDNA3.0-Flag expression vector using HindIII and XhoI recognition sequences. TRIM28 mutant plasmids (R487E, V488E) and truncated plasmids (TRIM28-C, TRIM28-N) were generated with the Q5 Site-Directed Mutagenesis Kit (NEB, E0552S) and stored in our laboratory. cDNA encoding the full-length Nsp from the HP-PRRSV strain was subcloned into the pCAGGS-HA expression vector. Primers for all constructs are listed in Table 1. *E. coli* strain DH5α was employed for transformation. Plasmid overexpression was achieved using Lipofectamine 2000 reagent (Invitrogen, 11668-019) following the manufacturer’s instructions.

**TABLE 1:** Primers used for PCR amplification

Primer name	Primer sequence (5′−3′)
H-TRIM28-F	ATGGCGGCCTCCGCGGCGGCAGCCT
H-TRIM28-R	GGGGGCCATCACCAGGGCCAC
TRIM28 R487E,V488E- F	GAAGAGAGCCTTGAACGCCTG
TRIM28 R487E,V488E- R	TGGCACCTTGCGCATAAGGCC
TRIM28-1-376-F	CCCAAGCTTATGGCGGCCTCCGCGGCGGCA
TRIM28-1-376-R	CCGCTCGAGTCAGAGGGCCCGGTGCAGCTG
TRIM28-377-835-F	CCCAAGCTTATGAAGATGATTGTGGATCCC
TRIM28-377-835-R	CCGCTCGAGTCAGGGGCCATCACCAGGGCC
HA-Nsp1α-F	GGAATTCATGTCTGGGATACTTGATCG
HA-Nsp1α-R	AGAGCTCCATAGCACACTCAAAAGGGC
HA-Nsp1β-F	GGAATTCGCTGACGTCTATGACATTGA
HA-Nsp1β-R	CGAGCTCACCGTACCACTTATGACTGC
HA-Nsp3-F	GGAATTCGGCCCACACCTCATTGCTGC
HA-Nsp3-R	CGAGCTCCTCAAGGAGGGACCCGAGCT
HA-Nsp4-F	GGGTACCGGCGCTTTCAGAACTCAAAA
HA-Nsp4-R	AAAACCCGGGTTCCAGTTCGGGTTTG
HA-Nsp5-F	GGAATTCGGAGGCCTTTCCACAGTTCA
HA-Nsp5-R	CGAGCTCCTCGGCAAAGTATCGCAAGA
HA-Nsp7α-F	GGAATTCTCGCTGACTGGTGCCCTCGC
HA-Nsp7α-R	CGAGCTCCTCCAGAACTTTCGGTGGGA
HA-Nsp7β-F	GGGTACCAACGGTCCCAATGCCTGG
HA-Nsp7β-R	CCCTCGAGTCATTCCCACTGAGCTCTTCTAT
HA-Nsp8-F	AGAATTCGCCGCCAAGCTTTCCGTG
HA-Nsp8-R	CGAGCTCGCAGTTTAAACACTGCTCCTTAGTC
HA-Nsp9-F	CGGGGTACCTTTAAACTGCTAGCCGCC
HA-Nsp9-R	GGAAGATCTTCACTCATGATTGGACCTGAG
HA-Nsp10-F	GGAATTCGGGAAGAAGTCCAGAATGTG
HA-Nsp10-R	GGGTACCTTCCAGGTCTGCGCAAATAG
HA-Nsp11-F	AGAATTCGGGTCGAGCTCCCCGC
HA-Nsp11-R	GGCTAGCTTCAAGTTGGAAATAGGCCG
HA-Nsp12-F	AGAATTCGGCCGCCATTTTACCTG
HA-Nsp12-R	GGGTACCATTCAGGCCTAAAGTTGGTT

### Confocal microscopy

Marc145, HEK293T, and HeLa cells were fixed with 4% paraformaldehyde at room temperature for 20 min and washed three times with phosphate buffered saline (PBS). Subsequently, the cells were incubated with 0.1% Triton X-100 on ice for 10 min and washed again with PBS. Following blocking with 5% FBS at room temperature for 30 min, the cells were incubated with primary antibodies (Table 2) for 1.5 h at room temperature and washed three times. They were then incubated with the corresponding secondary antibodies (Alexa-Fluor 488-conjugated and Alexa-Fluor 568-conjugated) in the dark for 1.5 h at room temperature and washed again. The cell nuclei were labeled with 4',6-diamidino-2-phenylindole (C1006, Beyotime) for 5 min and washed with PBS. Cells were examined using a confocal microscope (Nikon), and images were captured at 63× and 10× magnifications.

**TABLE 2:** Antibody information

Antibody	Source	Identifier
Anti-Beclin1 rabbit MAb	Abcam	ab207612
Anti-VPS34 rabbit MAb	CST	#4263
Anti-LC3 rabbit MAb	Sigma	L7543
Anti-TRIM28(KAP1) rabbit PAb	Proteintech	15202-1-AP
Anti-CRM1 mouse MAb	Proteintech	66763-1-lg
Anti-β-actin mouse MAb	Biodragon	B1029
Anti-Flag mouse MAb	Sigma	F1804
Anti-HA rabbit PAb	Amerbio	Ab1021t
Anti-His mouse MAb	Proteintech	66005-1-lg
Anti-LaminB1 mouse MAb	Biodragon	B1053
Anti-GFP mouse MAb	CMATAG	AT0028
Anti-PRRSV nucleocapsid (N) mouse PAb	Dr. Yihong Xiao, Shandong Agricultural University	N/A^*a*^
Peroxidase-conjugated Affinipure goat anti-mouse IgG	ZSGB-BIO	ZB2305
Peroxidase-conjugated Affinipure goat anti-rabbit IgG	ZSGB-BIO	ZB2301
Alexa Fluor 594 anti-Rabbit IgG	Proteintech	SA00006-4
Alexa Fluor 488 anti-Mouse IgG	Proteintech	SA00006-1

^*a*^ N/A, not available.

### Western blot analysis

Cells were washed twice with cold PBS and lysed in NP-40 buffer (Lysis Buffer 2×: NP-40: 1%; Hepes: 25 mM, pH 7.4; EDTA: 5 mM) containing a protease inhibitor mix (EDTA: 1 mM, pH 8.0; EGTA: 8 mM, pH 8.0; Na_3_VO_4_: 1 mM; NaF: 250 mM; PMSF: 100 µM). One part lysis buffer was mixed with one part protease inhibitor mix and 1 mM dithiothreitol (DTT) for cytoplasmic extracts, or cells were lysed in SDS lysis buffer (Tris-HCl: 100 mM, pH 6.8; 4% SDS; 0.2% bromophenol blue; 20% glycerol; 200 mM DTT) for total cell extracts. Cell lysates were boiled in a 6× loading buffer for 10 min, and equal amounts of samples were loaded onto 12% (wt/vol) SDS-PAGE gels. The separated proteins were transferred to methanol-activated polyvinylidene fluoride membranes (Millipore). Membranes were blocked with 5% BSA (Sigma) in PBS with Tween 20 (PBS-T) at room temperature for 1 h and incubated with various primary antibodies (Table 2) overnight at 4°C. After washing, the membranes were incubated with the appropriate secondary antibodies at room temperature for 1 h. Immunoreactive bands were visualized using an enhanced chemiluminescence (ECL) detection system (Tanon, 5100).

### Fractionation of nuclear and cytoplasmic extracts

First, 4×10^6^–8×10^6^ cells were harvested into 200 µL buffer A (Hepes: 10 mM; KCl: 10 mM; EDTA: 0.1 mM; EGTA: 0.1 mM) with protease inhibitor and DTT by gentle pipetting, and incubated on ice for 15 min. Four microliters of 10% NP-40 was added, followed by vortexing and incubation on ice for 2 min. The mixture was centrifuged at 10,000 rpm for 1.5 min at 4°C, and the supernatant, representing the cytoplasmic extract, was collected and boiled in buffer for 10 min. The pellet was lysed in 100 µL 1× SDS lysis buffer with 2.5% β-mercaptoethanol and heated at 95°C for 5 min, and used for subsequent protein level detection. The nuclear protein extraction Kit (A10039) is also used for the separation of nuclear proteins and cytoplasmic proteins. The separately collected nuclear protein and cytoplasmic protein samples are mixed with 5× SDS and heated at 95°C for 5 min for subsequent protein level detection. The abundance of TRIM28 in the nucleus and cytoplasm was detected by Western blotting.

### TaqMan fluorescent quantitative real-time PCR

Total RNA was extracted from cells using the Eastep Super Total RNA Extraction Kit (Promega, LS1040) following the manufacturer’s instructions. Reverse transcription was conducted with the M-MLV reverse transcriptase (TaKaRa, #2641A). TaqMan fluorescent RT-qPCR was performed on the ABI sequence detector system (StepOnePlus, Life Technologies Holdings Pte Ltd) in a final volume of 10 µL, including 2 µL cDNA (~100 ng/µL), 5 µL 2× UltraSYBR Mixture (High ROX) (CWBIO), 0.5 µL primer pairs, and 2 µL water. The PCR conditions were: denaturation at 95°C for 10 min, followed by 40 cycles of 95°C for 10 s, annealing at 55°C for 30 s, and extension at 72 ° C for 45 s. All primers used for quantitative real-time PCR are listed in Table 3. siRNA knockdown: Small interfering RNA (siRNA) sequences targeting TRIM28 (siTRIM28) and a negative control RNA (NC) were designed and synthesized by GenePharma (Table 4, Shanghai, China) using the Rosetta algorithm for siRNA design and NCBI BLAST for off-target analysis. Briefly, Marc145 cells in 12-well plates (60–80% confluence) were transfected with 15 µM siTRIM28 or NC using Lipofectamine RNAiMax (Invitrogen) for 24 h. Subsequently, the cells were infected with 0.1 MOI PRRSV and harvested at the indicated times. TRIM28 gene expression levels were confirmed by quantitative real-time PCR (qRT-PCR), and protein levels were verified by Western blotting.

**TABLE 3:** Primers used for qRT-PCR amplification

Primer name	Primer sequence (5′−3′)
TRIM28-F	ATGTGCTCCCTGACCTGAAG
TRIM28-R	CAGCAGAACACGCTCACATT
p-GAPDH-F	TACACTGAGGACCAGGTTGTG
p-GAPDH-R	TTGACGAAGTGGTCGTTGAG
mk-GAPDH-F	ACCCACTCTTCCACCTTCGACGCT
mk-GAPDH-R	TGTTGCTGTAGCCAAATTCG
PRRSV-N-F	GCCTCGTGTTGGGTGGCAGA
PRRSV-N-R	CACGGTCGCCCTAATTGAATAGG

**TABLE 4:** Primers used in small-interfering RNA assay

Primer name	Primer sequence (5′−3′)
Negative control	UUCUCCGAACGUGUCACGUTT
	ACGUGACACGUUCGGAGAATT
siTRIM28	GGACUACAACCUUAUUGUUTT
	AACAAUAAGGUUGUAGUCCTT

### Co-immunoprecipitations (Co-IP)

Marc145 and HEK293T cells cultivated in 10 cm dishes were transfected with the appropriate expression plasmid and harvested 24 h post-transfection. Following washing with cold PBS (pH 7.4), the cell pellet was lysed with NP-40 buffer (20 mM Tris, 150 mM NaCl, 1 mM EDTA, 1 mM EGTA, 1% NP-40, 2.5 mM sodium pyrophosphate, and 1 mM Na3VO4) containing 100 µM PMSF. Cell lysates were incubated with 2 µg antibody overnight at 4°C with gentle agitation. Subsequently, 20 µL of protein A/G PLUS-Agarose beads (Santa, sc-2003), pre-washed three times with cold PBS (pH 7.4), were added and incubated at 4°C for 4–6 h. The beads were washed three times with Co-IP lysis buffer, and the precipitates were eluted in 60 µL lysis buffer and 6× loading buffer by boiling at 95°C for 8 min. The supernatants were subjected to SDS-PAGE.

### Statistical analysis

Statistical significance was determined using a two-tailed Student’s *t*-test, with *P*<0.05 considered statistically significant.

## Data Availability

All data relevant to this work are contained within this article and its supplemental material.

## AFTER EPUB

*[This article was published on 24 September 2025 with incomplete Acknowledgments. The Acknowledgments were updated in the current version, posted on 25 September 2025.]*

## SUPPLEMENTAL MATERIAL

Supplemental figures jvi.01133-25-s0001.pdf Figures S1 to S3.

ASM does not own the copyrights to Supplemental Material that may be linked to, or accessed through, an article. The authors have granted ASM a non-exclusive, world-wide license to publish the Supplemental Material files. Please contact the corresponding author directly for reuse.
