# Supplementary material for: Cytoplasmic translocation of tripartite motif-containing 28 is critical for PRRSV-induced autophagy through promoting Vps34-Beclin1 complex formation
Source: J Virol. 2025 Sep 24;99(10):e01133-25. doi: 10.1128/jvi.01133-25 (PMC12548422; doi:10.1128/jvi.01133-25)
Supplement: Supplemental figures — Figures S1 to S3. [file jvi.01133-25-s0001.pdf]

Supplementary materials

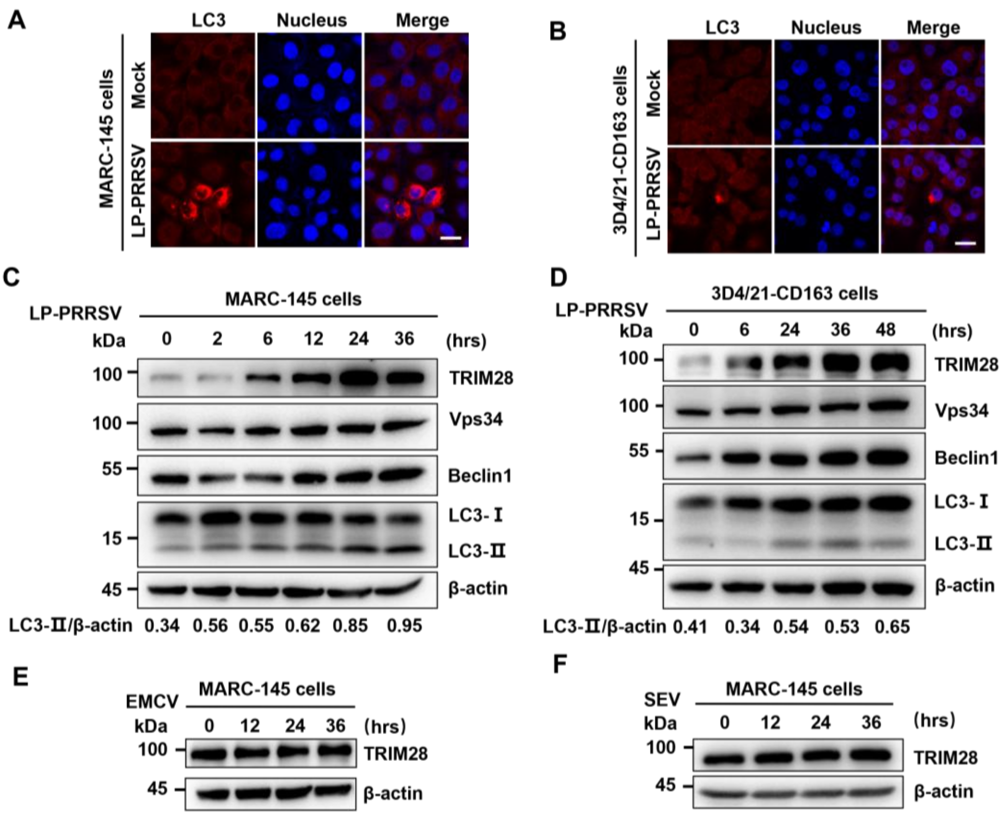

Figure S1. Correlation between PRRSV-induced autophagy and TRIM28, related to Figure 1. (A and B) Confocal microscopy analysis of LC3 puncta in Marc-145 (A) and 3D4/21 (B) cells infected with LP-PRRSV (MOI = 0.1) for 36 hours. Scale bars, 20 μm. (C and D) Immunoblot analysis of TRIM28, LC3, Beclin1, VPS34, and N-Pr in NP-40 cell lysates of Marc-145 (C) and 3D4/21 (D) cells infected with LP-PRRSV (MOI = 0.1) for the indicated times. (E and F) Immunoblot analysis of TRIM28 in Marc-145 cells infected with EMCV (E) or SEV (F) for the indicated times.

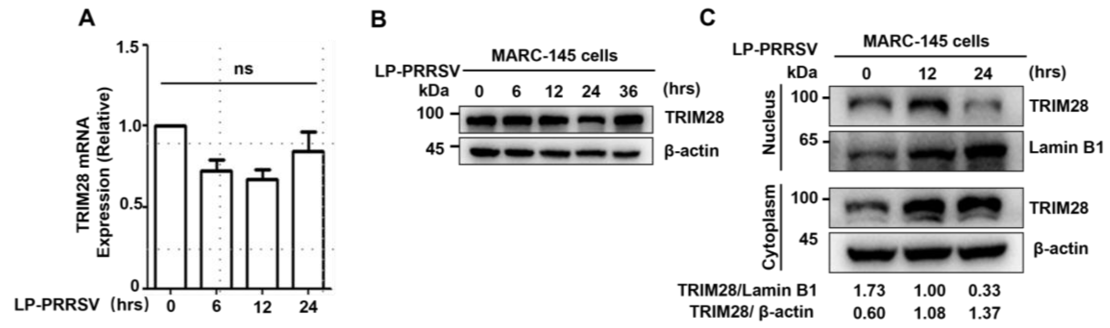

Figure S2. Transfer of TRIM28 from the nucleus to the cytoplasm under PRRSV infection, related to Figure 2. (A) qPCR analysis of TRIM28 in Marc145 cells infected with LP-PRRSV (MOI = 0.1) for the indicated times. (B) Immunoblot analysis of TRIM28 in whole-cell lysates of Marc145 cells infected with LP-PRRSV (MOI = 0.1) for the indicated times. (C) Immunoblot analysis of TRIM28 in cytoplasmic and nuclear fractions of Marc145 cells infected with LP-PRRSV for the indicated time points.

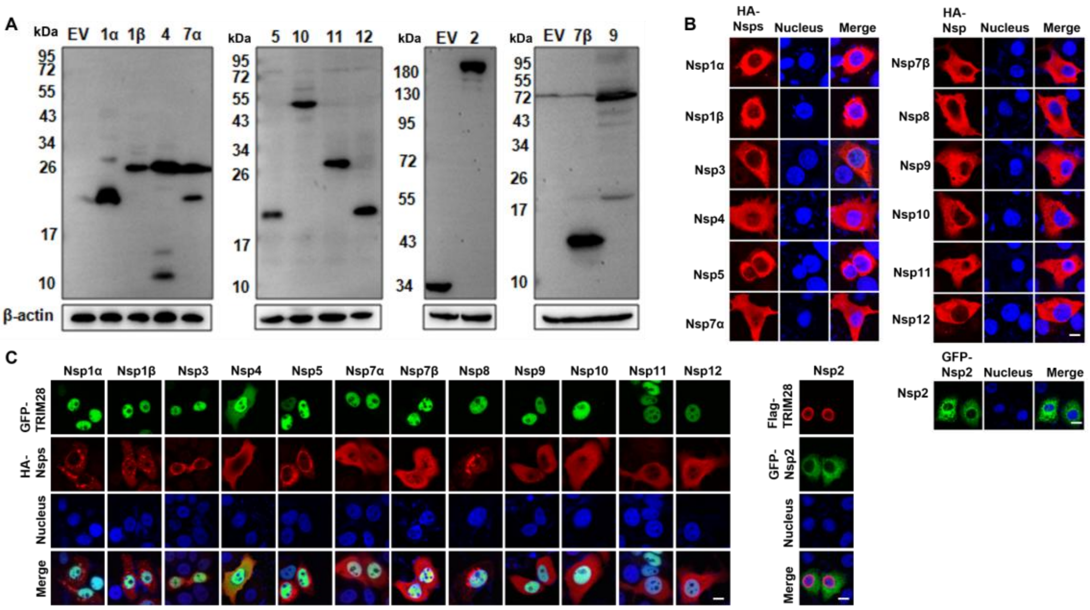

Figure S3. TRIM28 primarily colocalizes with PRRSV Nsp4 in the cytoplasm. (A, B) PRRSV nonstructural proteins were transfected into HEK-293T (A) and Marc145 (B) cells for 24 hours. Western blotting (A) and immunofluorescence (B) were used to detect protein expression. (Red indicates the location of the nonstructural protein, green indicates the location of Nsp2, and blue indicates the nuclear position stained by DAPI). Scale bars, 5 µm. (C) Co-localization of GFP-TRIM28 (Flag-TRIM28) with HA-tagged PRRSV nonstructural protein (GFP-Nsp2) in HeLa cells, examined by immunofluorescence confocal microscopy. Nuclei were stained with DAPI. Scale bars, 5 µm.
